# Supplementary material for: Perceptions of Nut Consumption amongst Australian Nutrition and Health Professionals: An Online Survey
Source: Nutrients. 2022 Apr 15;14(8):1660. doi: 10.3390/nu14081660 (PMC9032339; doi:10.3390/nu14081660)
Supplement: Supplementary file 1 [file nutrients-14-01660-s001.zip › nutrients-1644965-supplementary.pdf]

Supplementary Material

Supplementary Material S1

UNIVERSITY OF WOLLONGONG AUSTRALIA

PIS

Participant Information Sheet

This is an invitation to participate in a study conducted by researchers at the University of Wollongong and University of Otago. The purpose of this research is to explore knowledge, beliefs, and perceptions regarding nut consumption among health professionals and practitioners. We are also interested in how these factors influence recommendations made to patients regarding nut consumption. This research aims to help us understand health professionals' perceptions of eating nuts, and reasons that health professionals may or may not recommend patients include nuts in their diet.

We estimate that completing the survey will take approximately **10 - 15 minutes** of your time. Apart from the time to complete the survey, we can foresee no risks for you. Your involvement in the study is **voluntary and anonymous** and you are free to discontinue participation at any point whilst completing the survey.

In recognition of your contribution to the research, by completing the survey you will have the option to go into the draw to win **one of five $50 gift vouchers**. After completion of the survey, you will be invited to send an email for inclusion in the prize draw.

For further information regarding this survey, click here

By clicking **NEXT** you are agreeing to participate in this survey.

Thank you for your interest in this study.
Elizabeth Neale, Rachel Brown, and Georgie Tran

Survey requirements

To take part in the survey, it is required you are:

- A health professional or practitioner
- Located in Australia

If you meet the above criteria and would like to take part in the survey, please agree to the following terms and click **NEXT**.

You must select the statement below.

☐ I am a health professional or practitioner located in Australia.

Alternatively, you may exit the survey now if you do not wish to participate.

Part 1 - demographics

This part of the survey consists of questions relating to participant demographics, including your gender, age, and professional qualifications.

What is your gender?

- ○ Male
- ○ Female
- ○ ________ Other (please specify)
- ○ I prefer not to say

What is your age?

- ○ 18-24
- ○ 25-34
- ○ 35-44
- ○ 45-54
- ○ 55-64
- ○ 65 and older
- ○ I prefer not to say

What is the highest level of education you have completed?

- ○ Never attended school
- ○ High school up to year 10
- ○ High school up to year 11 or 12
- ○ Certificate / diploma e.g. Certificate IV in Allied Health Assistance, Diploma of Health Science
- ○ Bachelor degree (including Honours) e.g. Bachelor of Nutrition & Dietetics (Honours), Bachelor of Nursing
- ○ Post-graduate degree (Coursework and research degrees) e.g. Master of Nutrition & Dietetics, PhD, Doctor of Medicine
- ○ ________ Other (please specify)

What is your profession? (please select all that apply)

- ☐ Dietitian
- ☐ General Practitioner

- ☐ Registered nurse
- ☐ Nutritionist
- ☐ Naturopath
- ☐ Personal trainer
- ☐ ________ Other (please specify)

Have you received any nutrition or dietetic training?

- ○ Yes
- ○ No

What type of nutrition or dietetic training have you completed? (please select all that apply)

- ☐ Undergraduate degree
- ☐ Postgraduate degree
- ☐ Diploma or certificate
- ☐ Activities contributing to Continuing Professional Development (CPD) (please specify)
- ☐ Other (please specify)

How many years have you been working in your profession?

- ○ 0-2 years
- ○ 3-5 years
- ○ 6-10 years
- ○ 10-20 years
- ○ 20+ years

Part 2 description

The next part of the survey will ask about your perceptions of nuts and nut butters. For the purpose of this next section, please note:

The term "NUTS" refers to unprocessed nuts in their basic form (e.g. whole, raw, roasted). Foods that contain nuts as a major component such as muesli or nut bars are also included.

However, nut butters, coconut and seeds are NOT included as "nuts".

The term "NUT BUTTER" refers to spreads made mostly of nuts e.g. peanut butter, almond butter, but NOT Nutella or similar confectionary spread.

Please click **NEXT** to continue.

Part 2 - perceptions of nuts

What do you think about nuts or nut butters (in general)?

	Strongly agree	Agree	Neither agree nor disagree	Disagree	Strongly disagree	I don't know
They are healthy	○	○	○	○	○	○
They are low in energy/calories	○	○	○	○	○	○
They are high in protein	○	○	○	○	○	○
They are low in vitamins and minerals	○	○	○	○	○	○
They are high in fat	○	○	○	○	○	○
They are low in fibre	○	○	○	○	○	○
They are high in antioxidants	○	○	○	○	○	○
They are naturally high in salt/sodium	○	○	○	○	○	○
Some of them are high in selenium	○	○	○	○	○	○

What do you think about nuts or nut butters (in general) (continued)?

	Strongly agree	Agree	Neither agree nor disagree	Disagree	Strongly disagree	I don't know
Some of them are high in iron	○	○	○	○	○	○
They are filling	○	○	○	○	○	○
Eating them will cause people to gain weight	○	○	○	○	○	○
Eating them can INCREASE people's total blood cholesterol	○	○	○	○	○	○
Eating them can INCREASE people's risk of cardiovascular disease	○	○	○	○	○	○
Eating them can help LOWER people's risk of diabetes	○	○	○	○	○	○

Part 2 - perceptions of nuts

How would you rate the healthiness of unsalted peanuts compared to unsalted tree nuts (e.g. almonds, walnuts, etc.)? (Please select one)

Much more healthy	Slightly more healthy	About the same	Slightly less healthy	Much less healthy
○	○	○	○	○

Comments (if any):

Part 2 - perceptions of nuts

How much do you agree or disagree with the following statements?

	Strongly agree	Somewhat agree	Neither agree nor disagree	Somewhat disagree	Strongly disagree
Nuts are healthy and do not cause weight gain	○	○	○	○	○
Nuts are healthy but only in moderation	○	○	○	○	○
Nuts are not healthy as they are high in calories and fat	○	○	○	○	○
Eating nuts will cause weight gain	○	○	○	○	○

Part 2 - perceptions of nuts

Are you aware of any recommendations or dietary guidelines for nut consumption?

- ○ Yes
- ○ No

Part 2 - perceptions of nuts

What do you think are the current recommendations for nut consumption?

- ○ Nuts should be included in the diet daily
- ○ Nuts should be eaten in moderation
- ○ Nuts should be limited in the diet
- ○ Nuts should not be included in the diet
- ○ Other (please specify)

Part 2 - perceptions of nuts

What do you consider a standard serving size of nuts per day? (please select at least one unit of choice and type in the box provided)

- [ ] ______ Whole nuts
- [ ] ______ Handfuls
- [ ] ______ Cups
- [ ] ______ Tablespoons
- [ ] ______ Teaspoons
- [ ] ______ Grams
- [ ] ______ Other (please specify)
- [ ] I don't know

Part 2 - perceptions of nuts

What do you know about activated nuts (please select all that apply)?

- [ ] Activated nuts are healthier than regular nuts
- [ ] Regular nuts are healthier than activated nuts
- [ ] Activated nuts are better for digestion
- [ ] Activated nuts are similar in nutritional value to regular nuts
- [ ] I do not know much about activated nuts
- [ ] Other (please specify)

Part 3 - client recommendations

In your current or previous roles, have you ever discussed food with your patients or clients, or provided dietary advice to patients or clients?

- ○ Yes
- ○ No

Part 3 description

The next part of the survey will ask about whether or not you recommend nuts to some of your clients and patients (if applicable). Please answer to the best of your ability.

Please click **NEXT** to continue.

Part 3 - client recommendations

Do you give your clients advice regarding the consumption of nuts or nut butters? (Please select all that apply)

- ☐ Yes, I advise some of my clients to eat MORE
- ☐ Yes, I advise some of my clients to eat FEWER/LESS
- ☐ No, I do not mention eating them to my clients at all

Part 3 - client recommendations

What percentage of your clients or patients do you tell to eat **MORE** nuts or nut butters?

- ○ < 20%
- ○ 20-39%
- ○ 40-59%
- ○ 60-79%
- ○ 80-100%
- ○ I'm not sure

Part 3 - client recommendations

Of the clients who you tell to eat **MORE** nuts or nut butters, what percentage do you think follows your advice? (Please select one)

- ○ 0%
- ○ 1-19%
- ○ 20-39%
- ○ 40-59%
- ○ 60-79%
- ○ 80-100%
- ○ I'm not sure

Part 3 - client recommendations

When you advise your clients or patients to eat **MORE** nuts or nut butters, why do you do so? (Please select all that apply)

- ☐ They are good for health/nutritious
- ☐ They are a good source of energy/calories
- ☐ They are a good source of protein
- ☐ They are a good source of vitamins and minerals
- ☐ They are a good source of unsaturated fats

- [ ] They are a good source of fibre
- [ ] They are a good source of antioxidants
- [ ] Some of them are a good source of selenium
- [ ] Some of them are a good source of iron
- [ ] Eating them can help DECREASE risk of cardiovascular disease
- [ ] Eating them can help LOWER blood cholesterol
- [ ] Eating them can help promote satiety (fullness)
- [ ] Eating them can help with weight management
- [ ] Other (please specify)

Part 3 - client recommendations

Which type(s) of nuts do you advise your clients to consume regularly? (Please select all that apply)

- [ ] Almond
- [ ] Brazil
- [ ] Cashew
- [ ] Hazelnut
- [ ] Macadamia
- [ ] Peanut
- [ ] Pecan
- [ ] Pine nut
- [ ] Pistachio
- [ ] Walnut
- [ ] I recommend nuts in general
- [ ] ________ Other (please specify)

Part 3 - client recommendations

Which form(s) of nuts do you advise your clients to consume? (Please select all that apply)

- [ ] Raw
- [ ] Roasted with oil
- [ ] Roasted without oil
- [ ] Roasted unsalted
- [ ] Roasted and salted
- [ ] As part of a dish (e.g. satay sauce)

- [ ] Other (please specify)

Part 3 - client recommendations

How do you advise your clients to store their nuts? (Please select all that apply)

- [ ] In the original packaging
- [ ] In opaque (dark) containers
- [ ] In clear containers (e.g. clear tupperware, ziplock bags, etc.)
- [ ] In cupboards or pantries
- [ ] On counters or desks
- [ ] In an open container or bowl
- [ ] In paper bags
- [ ] In the freezer
- [ ] I don't provide advice on how to store nuts
- [ ] Other (please specify)

Part 3 - client recommendations

Do you provide your clients with recipes on how to incorporate nuts into meals?

- ○ Yes
- ○ No

Please provide the name of ONE recipe you provide clients with to help them incorporate nuts into meals

Part 3 - client recommendations

Which type(s) of nut butter do you advise your clients to consume regularly? (Please select all that apply)

- [ ] Almond butter
- [ ] Cashew butter

- ☐ Hazelnut butter
- ☐ Peanut butter
- ☐ Walnut butter
- ☐ I recommend nut butters in general
- ☐ I do not recommend my clients consume nut butters
- ☐ Other (please specify)

Part 3 - client recommendations

What percentage of your clients or patients do you tell to eat **FEWER** nuts or nut butters?

- ○ < 20%
- ○ 20-39%
- ○ 40-59%
- ○ 60-79%
- ○ 80-100%
- ○ I'm not sure

Part 3 - client recommendations

Of the clients who you tell to eat **FEWER** nuts or **LESS** nut butters, what percentage do you think follows your advice?

- ○ 0%
- ○ 1-19%
- ○ 20-39%
- ○ 40-59%
- ○ 60-79%
- ○ 80-100%
- ○ I'm not sure

Part 3 - client recommendations

When you advise your patients or clients to eat **FEWER** nuts or **LESS** nut butters, why do you do so? (Please select all that apply)

- ☐ They are unhealthy
- ☐ They are high in energy/calories
- ☐ They are high in fat

- [ ] They are naturally high in salt/sodium
- [ ] Regular consumption of them can INCREASE risk of cardiovascular disease
- [ ] Regular consumption of them can INCREASE blood cholesterol
- [ ] Regular consumption of them can cause weight gain
- [ ] There is conflicting information and I do not want to confuse my clients or patients
- [ ] There is contraindication(s) with their medication
- [ ] They are too expensive for my clients
- [ ] My clients have dental issues, making it inconvenient/uncomfortable for them
- [ ] My clients have more pressing concerns than nut consumption
- [ ] I am concerned about nut allergy
- [ ] I do not know enough about nuts and their benefits
- [ ] Other (please specify)

Part 3 - client recommendations

What amount of nuts or nut butters do you advise clients to consume? (please select at least one unit of choice and type in the box provided)

- [ ] ______ Whole nuts
- [ ] ______ Handfuls
- [ ] ______ Cups
- [ ] ______ Tablespoons
- [ ] ______ Teaspoons
- [ ] ______ Grams
- [ ] ______ Other (please specify)
- [ ] I don't know

Part 3 - client recommendations

When you advise your clients to eat nuts or nut butters, how often do you tell them they should do so?

- ○ Every day
- ○ 5+ times per week
- ○ 2-4 times per week
- ○ Once per week
- ○ Several times per month

- [ ] ◯ Once or less than once per month

Part 3 - client recommendations

Why do you choose to not mention nut consumption to your clients **AT ALL**? (Please select all that apply)

- [ ] Nuts are unhealthy
- [ ] Nuts are high in energy/calories
- [ ] Nuts are high in fat
- [ ] Nuts are naturally high in salt/sodium
- [ ] Regular consumption of nuts can INCREASE risk of cardiovascular disease
- [ ] Regular consumption of nuts can INCREASE blood cholesterol
- [ ] Regular consumption of nuts can cause weight gain
- [ ] There is conflicting information and I do not want to confuse my clients or patients
- [ ] There is contraindication(s) with their medication
- [ ] My clients or patients have dental issues, making it inconvenient/uncomfortable for them
- [ ] My clients have more pressing concerns than nut consumption
- [ ] Nuts are too expensive for my clients
- [ ] I am concerned about nut allergy
- [ ] I do not know enough about nuts and their benefits
- [ ] I do not think my advice would cause a change in their eating behaviour
- [ ] Other (please specify)

Part 3 - client recommendations

During your sessions, do your clients provide reasons for not eating nuts or nut butters?

- ◯ Yes, MOST of my clients do
- ◯ Yes, SOME of my clients do
- ◯ Yes, A FEW of my clients do
- ◯ No, my clients do not

Part 3 - client recommendations

What are the reasons most frequently provided by your clients for not eating more nuts, or for not eating nuts at all? (Please select all that apply)

- [ ] Nuts are unhealthy

- ☐ Nuts are high in energy/calories
- ☐ Nuts are high in fat
- ☐ Nuts are naturally high in salt/sodium
- ☐ Eating nuts can INCREASE the risk of cardiovascular disease
- ☐ Eating nuts can INCREASE blood cholesterol
- ☐ Eating nuts can cause weight gain
- ☐ They dislike the taste and/or smell
- ☐ They dislike the texture
- ☐ They are allergic to nuts
- ☐ They are nut intolerant
- ☐ They live with / are in close contact with someone who is allergic to nuts
- ☐ They have dental issues, making it inconvenient/uncomfortable for them
- ☐ They have more pressing concerns than nut consumption
- ☐ Nuts are too expensive
- ☐ They are unsure how to include nuts in meals/recipes
- ☐ There is no supply / nuts are difficult to purchase
- ☐ Other (please specify)

Part 4 description

The final part of the survey will ask about your interest in further training or education resources relating to nuts.

Please click **NEXT** to continue.

Part 4 - further education

If accessible, would you be interested in taking part in training courses or receiving education resources relating to nuts?

- ○ Yes
- ○ No
- ○ I'm not sure

Part 4 - further education

What form of training would you be interested in? (please select all that apply)

- ☐ Written education resources (for health professionals)

- [ ] Webinars
- [ ] Client education resources
- [ ] Face-to-face workshop
- [ ] Podcasts
- [ ] Summaries of current scientific evidence
- [ ] Other (please specify)

Final comments

Do you have any other comments regarding nuts?

Final comments

Do you have any general feedback or recommendations for this survey?

Powered by Qualtrics

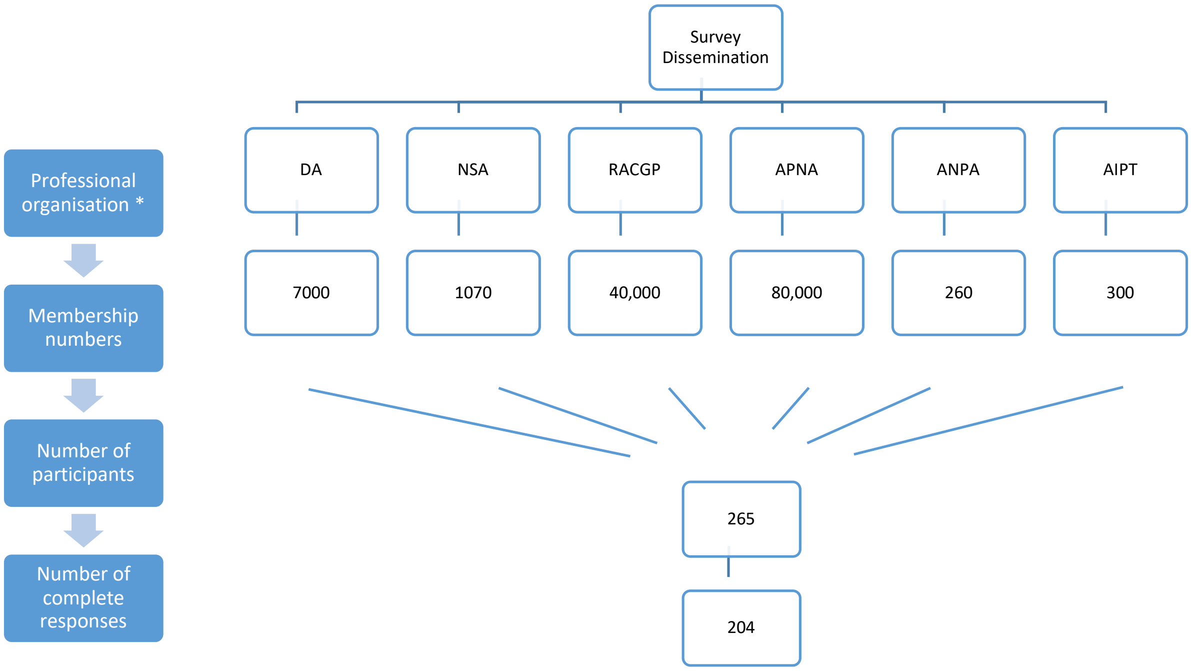

Supplementary Material S2: Breakdown of survey disseminations to each professional organisation and number of responses received.

* Professional organisations: DA = Dietitians Australia, NSA = Nutrition Society of Australia, RACGP = Royal Australian College of General Practitioners, APNA = Australian Primary Health Care Nurses Association, ANPA = Australian Naturopathic Practitioners Association, AIPT = Australian Institute of Personal Trainers.

Supplementary Material S3: Responses from health professionals *n* (%) regarding the current recommendations for nut consumption.

Recommendation	All health professionals (*n* = 163^)	Dietitians / Nutritionists (*n* = 148)	Non-Dietitians / Nutritionists (*n* = 15)	*p*-value
**Nuts should be included in the diet daily**	117 (72)	109 (74)	8 (60)	0.130
**Nuts should be eaten in moderation**	40 (25)	34 (23)	6 (40)	0.204
**Nuts should be limited in the diet**	0 (0)	0 (0)	0 (0)	n/a #
**Nuts should not be included in the diet**	0 (0)	0 (0)	0 (0)	n/a #
**30g of nuts per day***	4 (2)	4 (3)	0 (0)	n/a #

^Only 163 out of 204 health professionals answered yes to being aware of recommendations for nut consumption. *p*-value for differences between health professionals calculated by Fisher's Exact test. * indicates free text response that participants submitted, and not an option that participants could choose from in the survey. # indicates no calculated *p*-value as there were no responses recorded in one or more of the groups.

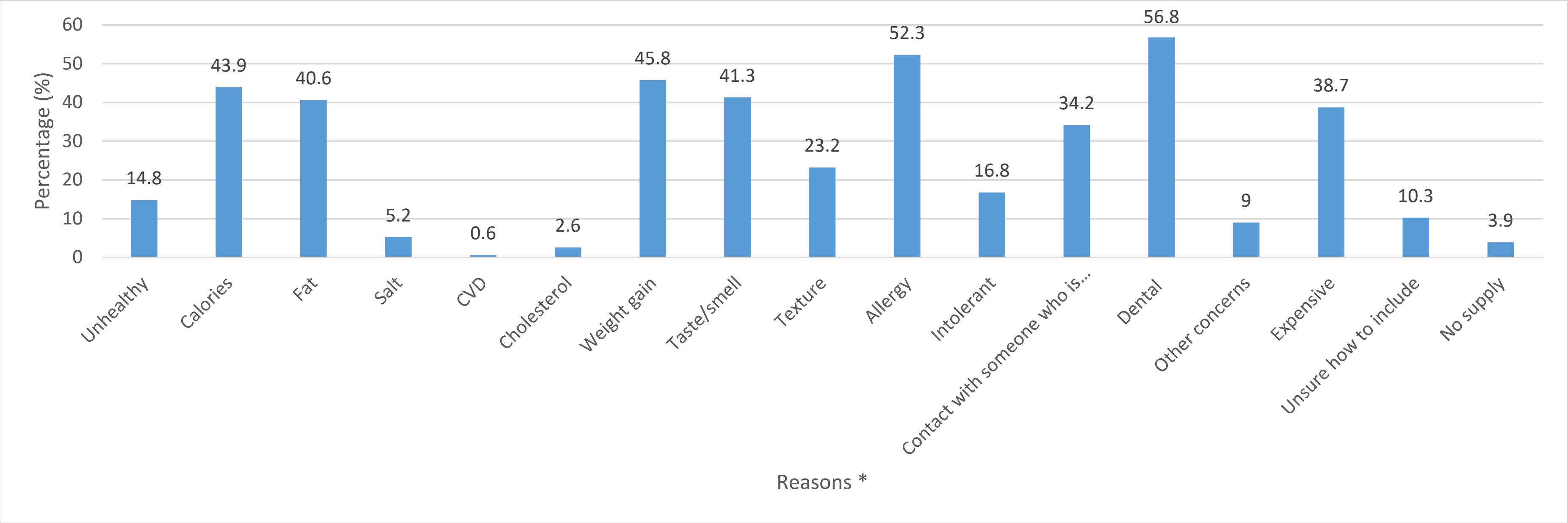

Supplementary Material S4: Reasons most frequently provided by clients to health professionals for not eating more nuts, or for not eating nuts at all.

* Figures were based on the percentage of health professionals who selected the following reasons provided by clients: Unhealthy = nuts are unhealthy, Calories = nuts are high in energy / calories, Fat = nuts are high in fat, Salt = nuts are naturally high in salt / sodium, CVD = eating nuts can increase risk of cardiovascular disease, Cholesterol = eating nuts can increase blood cholesterol, Weight gain = eating nuts can cause weight gain, Taste / smell = they dislike the taste and / or smell, Texture = they dislike the texture, Allergy = they are allergic to nuts, Intolerant = they are nut intolerant, Contact with someone who is allergic = they live with / are in close contact with someone who is allergic to nuts, Dental = they have dental issues, making it inconvenient / uncomfortable for them, Other concerns = they have more pressing concerns than nut consumption, Expensive = nuts are too expensive, Unsure how to include = they are unsure of how to include nuts in meals / recipes, No supply = there is no supply / nuts are difficult to purchase. Figures were based on the 155 out of 204 health professions who had clients provide reasons for not eating more nuts, or for not eating nuts at all.
